# Study adsorbents based on bent-Al_13_-CS-CTA and its application to the removal of CR from wastewater

**DOI:** 10.1039/d4ra00197d

**Published:** 2024-04-26

**Authors:** Hanjie Chen, Mei Zhang, Shuyang Chen, Ying Fang

**Affiliations:** a Materials Science and Engineering Department, Nanjing Tech University Nanjing 210000 China chenhanjie@njtech.edu.cn

## Abstract

For rapid and efficient removal of Congo red (CR) from aqueous solutions, a composite of bent-Al_13_-CS-CTA was prepared from bentonite (bent), chitosan (CS), citric acid (CTA) and Al_13_ compounds. To comprehend the adsorption process, adsorption variables were changed, including initial pH of the solution, contact time, temperature, initial CR concentration, and adsorption dose. Bent intercalated with X-ray diffraction (XRD), specific surface area (BET), scanning electron microscopy (SEM) and Fourier transform infrared spectrophotometry (FTIR) were used to analyze the material. Physicochemical and structural analysis proven the incorporation of Al_13_, CS, and CTA into the bent matrix. The pseudo-second-order model aligns with the adsorption kinetics. The adsorption isotherm conformed to the Langmuir adsorption isotherm, with a maximum adsorption capacity of 476.8 mg g^−1^ at pH 9, a dosage of 2 g L^−1^, and a temperature of 25 °C. Upon examining the thermodynamic properties of Δ*S*, Δ*H*, and Δ*G*, it was found that the reaction is a spontaneous endothermic process that could potentially be utilized to eliminate CR.

## Abstract 2

For rapid and efficient removal of Congo red (CR) from aqueous solutions, a composite of bent-Al_13_-CS-CTA was prepared from bentonite (bent), chitosan (CS), citric acid (CTA) and Al_13_ compounds.

## Graphical abstract

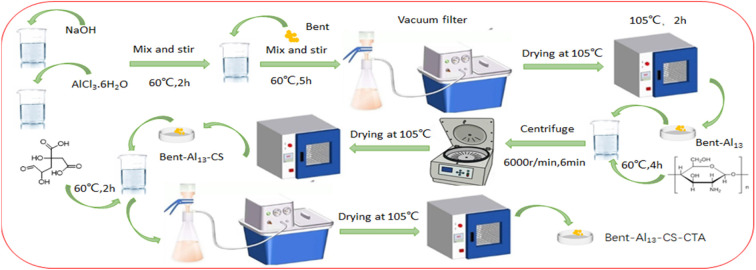

## 1. Introduce

Since the second half of the 20th century, industrialization and urbanization have grown exponentially, and, as a result, an abundance of synthetic dyes are being widely employed in a comprehensive range of industrial production and processing.^1^ Among these dyes, Congo red (CR)^2^ is a representative anionic azo dye that is commonly used in an extensive range of industrial sectors, such as pulp, paper, paints, textiles, cosmetics, and pharmaceuticals. Thanks to the presence of azo bonds and sophisticated aromatic rings, it has carcinogenic, genotoxic, and mutagenic impacts. Meanwhile, the non-biodegradability,^3,4^ high water solubility and intense coloration of CR makes their removal from contaminated water a demanding.^5^ Due to untreated or improperly handled CR solution, it is inevitable that a multitude of CR will be discharged into the water body, which damages the aquatic ecosystem and its inhabitants, resulting in catastrophic consequences including congenital toxicity, skin sensitization,^6^ irritation, and the obstruction of sunlight for aquatic flora.^7^

Currently, the disposal methods for CR are categorized into physical, chemical, and biological approaches. These methods include flocculation,^8^ ion exchange,^9^ photocatalysis,^10^ adsorption,^11–13^ membrane filtration,^14^ and electrochemistry.^15^ These technologies have been used to treat water contaminated with CR dyes. Whereas chemical and biological methods are highly effective for removing, they also generate numerous by-products. Adsorption is particularly effective in removing smelly and eliminating CR. It is possible to remove CR at significantly low concentrations, and the water quality remains stable after treatment, without any secondary pollution. Universally used adsorption materials include mineral clays,^16^ natural organic chitosan, cellulose,^17^ and activated carbon. Nevertheless, the cost of producing activated carbon remains high, which limits its usage in developing countries and renders it unsuitable for commercialization.

Bent,^18^ a natural mineral material with montmorillonite as its main component, possesses high thermal stability and a large sizable surface area on ground of its unique chemical composition and special structure.^19^ It is a cost-effective adsorbent that is readily available in diverse countries and is commonly used to treat CR dye wastewater as an environmentally friendly material. By virtue of its structural characteristics, it exhibits limited effectiveness in removing the organic anionic dye CR. It is difficult to separate solids and liquids, which is easy to cause material waste and secondary pollution.^20^ However, by increasing the interlayer spacing, and optimizing the aperture channels, the treatment efficiency for CR was significantly enhanced. AlCl_3_·6H_2_O was utilized to hydrolyze metal salts in an alkaline solution to obtain an intercalation solution for preparing Al columnar bentonite, thereby improving the basal layer spacing. Fadhila Ayari *et al.*^21^ conducted a study on the treatment of Congo red dye in aqueous solution using Al/Fe pillared bentonite. The results of their study indicate that the various synthesized adsorbents have a higher adsorption capacity for CR compared to the original bentonite. Chitosan (CS), a biodegradable hydrophilic polymer, is non-toxic and possesses various functional groups, including amino and hydroxyl groups, which give it excellent dye adsorption capacity.^22,23^ CS molecular chains penetrate the interlayer of columnar bent and attach to its surface, strengthening its adsorption capacity. Chong Huang *et al.*^24^ looked into the adsorption effect of acid–chitosan–bentonite composite on Cr^6+^ in solution. Their findings suggest that the maximum adsorption capacity may be 111.47 mg g^−1^ and that spontaneous and endothermic monolayer chemical adsorption is the adsorption mechanism. Citric acid is a biodegradable, non-toxic small molecule organic acid that can be extracted from natural biomass at a low cost and is readily available due to abundant reserves.^25^ In addition, the –OH and –COOH groups in CTA are not only effective linker groups for attaching CTA onto bentonite, but also serve as good chelating groups for removing organic dyes and heavy metals. Since bent is permanently electronegative, it will repel the anionic CR dye and dissolve it in citric acid (CTA)^26^ solution to change its electronegativity. A low-cost, environmentally friendly and recyclable adsorption material (bent-Al_13_-CS-CTA) with high efficiency was prepared by step inorganic–organic modification.^27^ The effects of operating parameters, such as adsorbent dosage, solution pH, contact time, and the initial concentration of CR dye on the adsorption efficiency, were investigated. Various kinetic models and isotherm models were employed to analyze the adsorption data. The possibility of reuse was thoroughly examined.

## 2. Materials and methods

### 2.1 Materials

The reagents utilized in the process include Congo red (C_32_H_22_N_6_Na_2_O_6_S_2_, 696.66), bentonite (100 mesh), chitosan (medium viscosity, 200–400 mPa s), citric acid (C_6_H_8_O_7_, 192.13), HCl, NaOH, AlCl_3_·6H_2_O and CH_3_COOH. These reagents were obtained from XL Technology Co., Ltd. and were used without further purification.

### 2.2 Synthesis of bent-Al_13_-CS-CTA

**Fig. 1:**
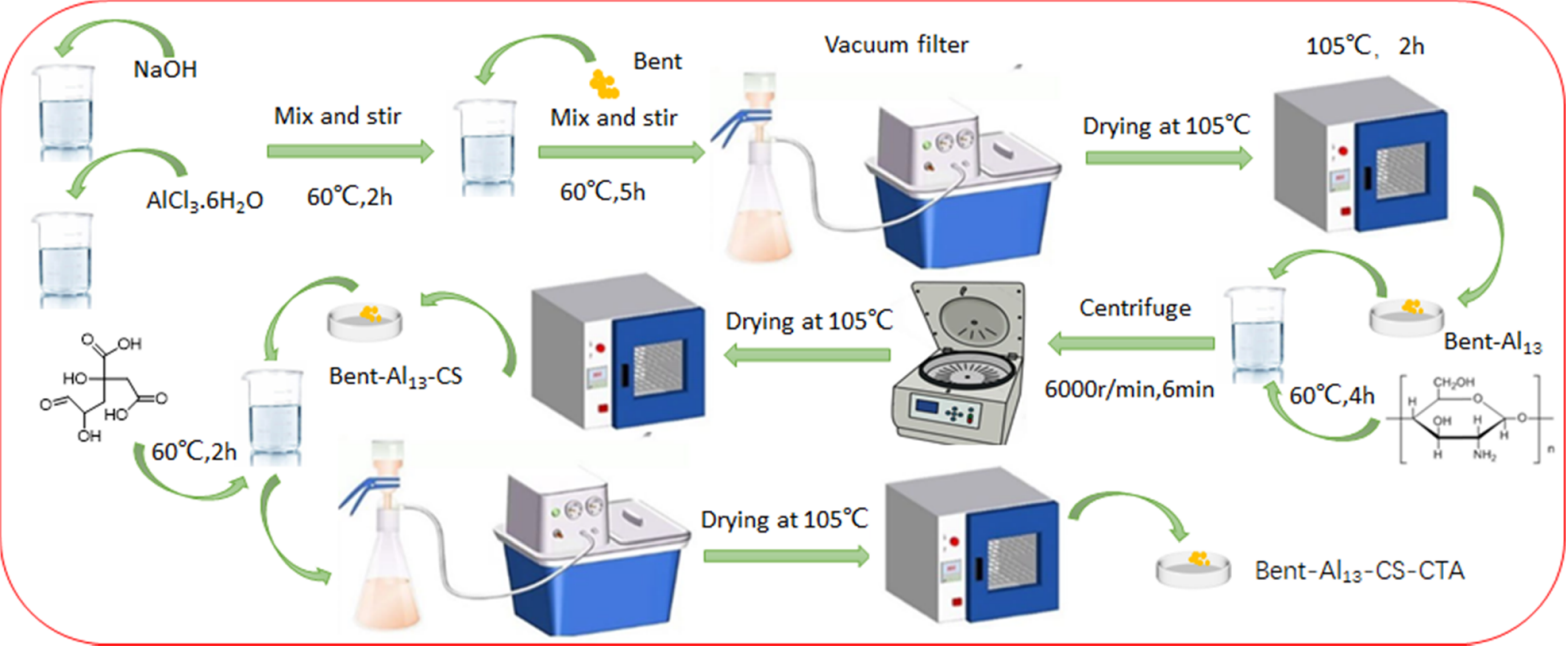
The synthesis process of bent-Al_13_-CS-CTA.

Firstly, a 0.5 M NaOH solution was gently added to a vigorously stirred 0.3 M AlCl_3_·6H_2_O solution at 60 °C, achieving a ratio of *n*[OH^−^]/*n*[Al^3+^] = 0.8.^28^ The stirring process continued for 2 hours, followed by incubation at an elevated temperature for 24 hours. Subsequently, the resulting mixture was then agitated for 5 hours at 60 °C and maintained at this temperature for another 24 hours before being rinsed multiple times with deionized water until no chloride ions were visible. After grinding, the mixture was set aside after adding it to the dry ingredients at 105 °C. Furthermore, a CS solution with a concentration of 2% was combined with the previously mentioned bent-Al_13_ powder in a mass ratio of *m*(CS)/*m*(bent-Al_13_) = 1/4. The resulting mixture underwent agitation for 4 hours before being subjected to centrifugation, purification, drying, and pulverization to yield bent-Al_13_-CS. Finally, a specific amount of bent-Al_13_-CS powder was added to the CTA solution in order to achieve a molar ratio of m (CTA)/*m*(ent-Al_13_-CS) = 2, resulting in the formation of bent-Al_13_-CS-CTA (Fig. 1).

### 2.3 Characterization methods

The functional groups of bent-Al_13_-CS-CTA were determined using a Thermo Scientific Nicolet iS20 Fourier transform infrared spectrophotometer. We studied the sample's morphological structure with a Hitachi Regulus 8100 scanning electron microscope. The samples were analysed using an X-ray diffractometer, the Smart Lab SE, with a tube voltage of 40 kV and a current of 30 mA. A Micromeritics 2460 specific surface area and porosity analyzer was used to measure the surface area and pore size of bent-Al_13_-CS-CTA.

### 2.4 CR adsorption studies

The adsorption of CR from an aqueous solution onto bent-Al_13_-CS-CTA was systematically investigated. A stock solution of CR (1500 mg L^−1^) was produced before the adsorption study. In order to obtain various concentrations of CR solution, dilute the stock solution previously prepared while maintaining a constant pH. A 722G spectrophotometer was used to measure the absorbance, which records the characteristic absorption peak of CR at 498 nm.^29^ The mixture was stirred on a stir plate at 600 rpm for 60 minutes. The study investigated the impact of varying dosages of bent-Al_13_-CS-CTA (0.5–3 g L^−1^), pH levels ranging from 3 to 11, contact durations from 10 to 150 minutes, initial CR concentrations ranging from 200 to 1400 mg L^−1^, and temperatures of 25 °C, 35 °C, and 45 °C on the effectiveness of the adsorbent. The adsorption efficiency (*R*) and the equilibrium adsorption capacity were calculated using eqn (1) and (2), respectively.1
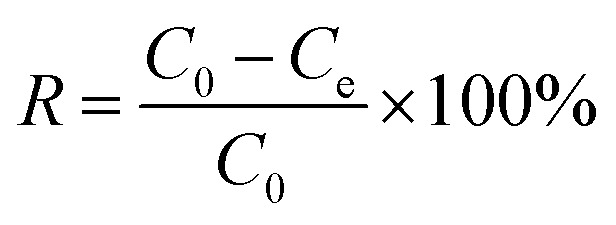
2
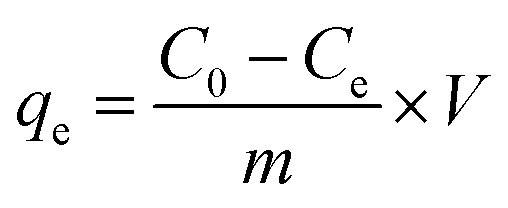
where *C*_0_ and *C*_e_ represent the initial and equilibrium concentrations (mg L^−1^) of CR, respectively, and *R* is the removal efficiency of the adsorbent. In addition, the adsorption capacities (mg g^−1^) at equilibrium time is denoted by *q*_e_. *m* and *V* refer to the mass of the adsorbed (*g*) and the volume of the dye taken (*L*) in turn.

## 3. Results and discussion

### 3.1 Characterization analysis

**Fig. 2:**
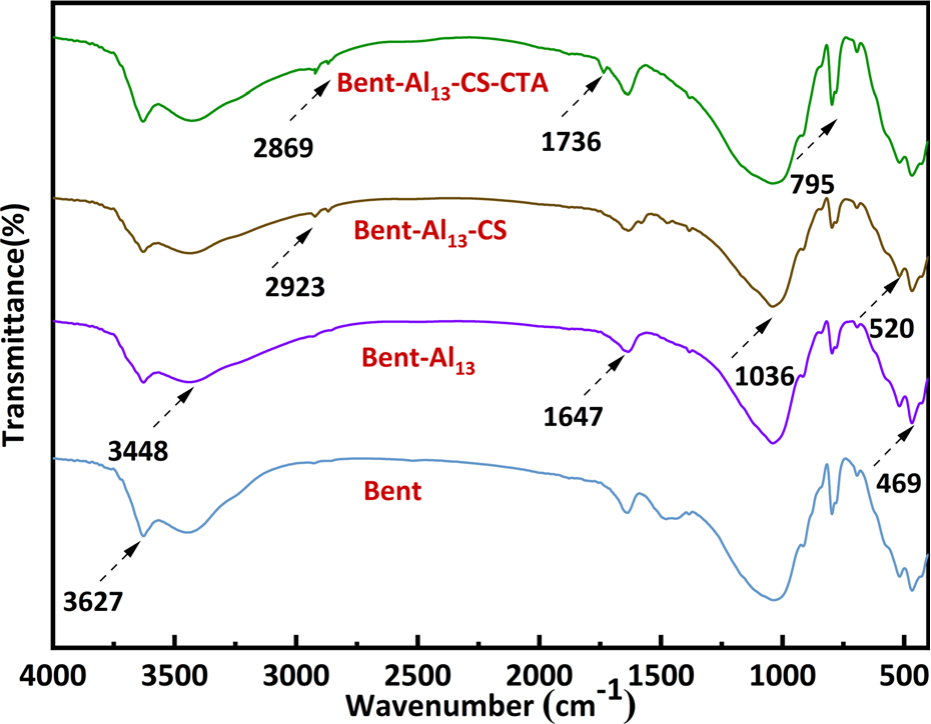
ATR-FTIR spectra of bent, bent-Al_13_, bent-Al_13_-CS and bent-Al_13_-CS-CTA.

Fig. 2 implies a strong absorption peak near 3627 cm^−1^, indicating the presence of crystalline water in the montmorillonite lattice. This peak is related to the telescopic vibration absorption peak of silicate Al–O–H.^30^ Simultaneously, there is a broad absorption peak near 3448 cm^−1^, which is characteristic of bent containing surface adsorbed water and interlayer structural water. The presence of water adsorbed between the layers of montmorillonite crystals can be inferred by observing stretching vibrations of –OH at 1643 cm^−1^. The stretching vibration absorption peak of Si–O–Si causes the absorption peak near 1024 cm^−1^. The bending vibration absorption peak of Si–O–Mg and Si–O–Fe is 520 cm^−1^ and 469 cm^−1^, sequentially.^30^ The absorption maxima at 2923 cm^−1^ and 2869 cm^−1^ are situated one at a time in the –CH symmetric and antisymmetric stretching vibrations of CS.^31^ These peaks manifest that CS is not only adsorbed on the surface but also penetrates into the interlayer of bent. Similarly, the peak at 1036 cm^−1^ became more pronounced, suggesting the presence of an electrostatic interaction between the surfaces of CS and bent. The appearance of a new peak at 1736 cm^−1^ demonstrates a symmetrical vibration characteristic of the carboxyl group, which suggests that the CTA has been successfully incorporated onto the surface of the bent. The stepwise processing of bent does not affect its basic structure and retains the characteristic peaks of bent.

**Fig. 3:**
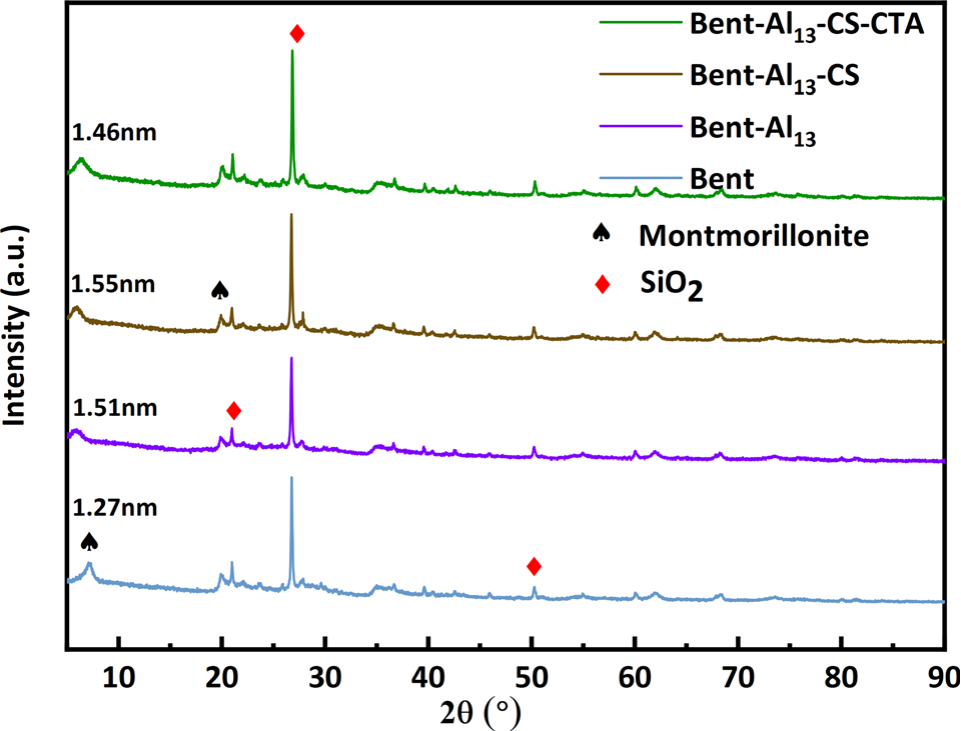
X-ray of bent, bent-Al_13_, bent-Al_13_-CS and bent-Al_13_-CS-CTA.

Based on the resultant XRD patterns (Fig. 3), the change in the crystalline phases at the various stages of the composite's synthesis was examined. The X-ray diffraction analysis showed that the main bent diffraction peaks at 7.03° and 19.87° corresponded to montmorillonite, while the peaks at 20.87°, 26.76°, and 50.24° at 2*θ* were primarily SiO_2_.^32^ The interlayer gap rises and the (001) diffraction peak shifts to a low angle with the addition of Al_13_, indicating the entry of Al_13_ into the interlayer. The layer spacings of bent, bent-Al_13,_ bent-Al_13_-CS, and bent-Al_13_-CS-CTA were 1.27 nm, 1.51 nm, 1.55 nm, and 1.46 nm, respectively, as determined by XRD diffraction circumstances and Bragg's formula: 2*d* sin*θ* = *nλ*. After adding CS to bent-Al_13_, some of it penetrated the bent-Al_13_ layer, while some of the load was on the surface of bent-Al_13_. The layer spacing rose from 1.27 nm to 1.55 nm, confirming that the montmorillonite layer swelled considerably under the action of Al_13_ and chitosan chains. The (001) diffraction peak moved to a low angle following the addition of CTA, most likely as a result of CTA dissolving the chitosan on the surface and dredging the bent-Al_13_-CS's interior channels, which facilitated the adsorption of CR in the aqueous solution. Infrared spectroscopy confirms that no modifications have been made to the fundamental structure.

Table 1 proves the porous structure parameters for the different samples, including specific surface area (*S*_BET_), external surface area (*S*_ext_), microporous surface area (*S*_μp_), microporous volume (*V*_μp_) and average pore diameter.^33^ It was observed that the specific surface of the bent and bent-Al_13_-CS-CTA increased from 69.88 m^2^ g^−1^ to 77.12 m^2^ g^−1^, and the micropore volume of the *t*-plots increased from 0.00136 m^3^ g^−1^ to 0.00149 m^3^ g^−1^ after modification with Al_13_, CS and CTA, which significantly improved the removal efficiency of CR. The specific surface area and micropore volume decreased after adsorption, indicating that CR molecules entered the micropores and were adsorbed onto the surface of the adsorbent. At the same time, scanning electron microscope (SEM) images can also support this claim. The increased average pore size also ensures consistent applicability.

**Table 1:** Comparison of the BET surface area and pore size of bent, bent-Al_13_-CS-CTA and bent-Al_13_-CS-CTA-CR

Sample	*S*_BET_ (m^2^ g^−1^)	*S*_ext_/*s*_μp_ (m^2^ g^−1^)	*V*_μp_ (m^2^ g^−1^)	*D* (nm)
Bent	69.88	40.08/29.8	0.0136	6.741
Bent-Al_13_-CS-CTA	77.12	42.45/34.67	0.0149	5.318
Bent-Al_13_-CS-CTA-CR	45.34	32.65/12.69	0.0053	6.751

**Fig. 4:**
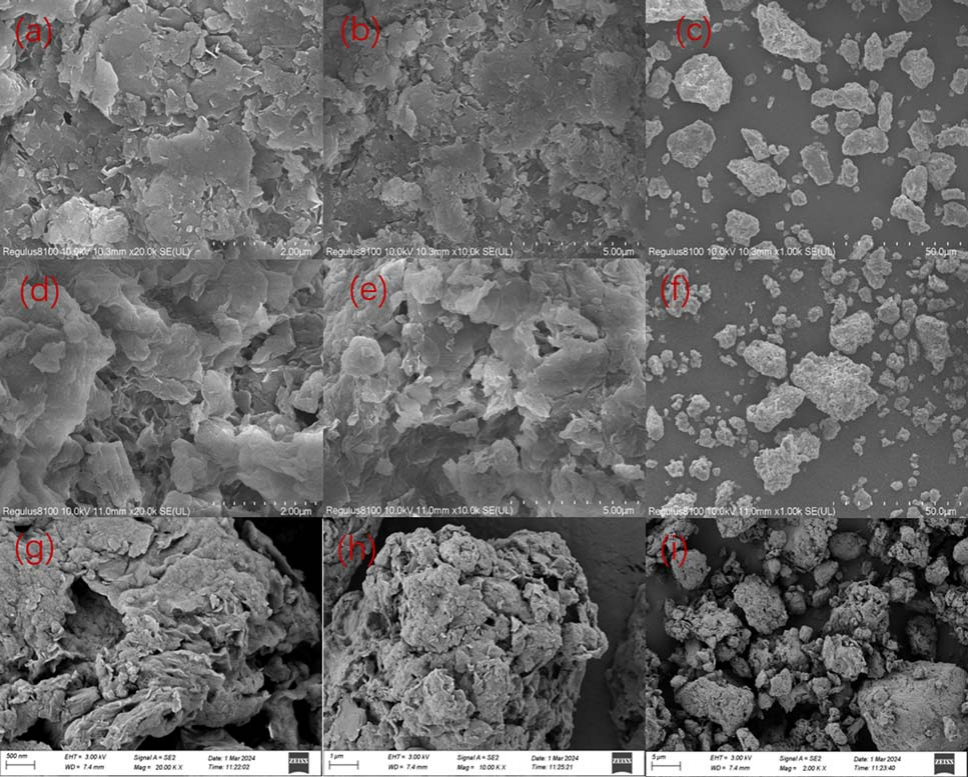
Scanning electron microscopy of bent ((a)–(c)), bent-Al_13_-CS-CTA((d)–(f)) and bent-Al_13_-CS-CTA-CR ((g)–(i)).

Fig. 4(a)–(c) clarify that bent has an irregular morphology, with obvious cracks on its surface, while the surface is smooth and the edges are clear.^34^ The modifier causes the initially dense mass of bent to become a loose structure with noticeable pores. The surface pores provide additional sites for dye molecules to penetrate and diffuse, facilitating the adsorption process,^35^ as shown in (d)–(f). Furthermore, the layer spacing is significantly increased, resulting in a rougher surface, smaller particles, and uneven dispersion, all of which enhance the composite's ability to absorb CR. After the adsorption is completed, as shown in Fig. 4(g)–(i), the layered structure of the adsorbent has not changed. There is a significant covering between layers and on the surface, leading to reduced channels and a dense arrangement of particles. This is because the Congo red molecule occupies the channels of the adsorbent and attaches between the layers.

### 3.2 Mechanism of the increased adsorption efficiency of the bent-Al_13_-CS-CTA

It is well known that the adsorption capacity of any adsorbent depends mainly on its physical and chemical properties, as well as the surface area of the adsorbent, the steric hindrance of bulk molecules, the adsorption site of the heterogeneous phase, and the interaction between the adsorbent and CR.^36^ The thermodynamic equation parameters indicate that this process is an endothermic reaction, and the increase in temperature enhances the adsorption reaction. Langmuir's equation fits well with the kinetics model and confirms that it represents a monolayer coverage. By increasing the layer spacing, chitosan molecules penetrate into the interlayer, and citric acid is loaded onto the bent surface, which the surface has been improved. Compared to bent, bent-Al_13_-CS-CTA provides a sufficient quantity of adsorption sites. Bent is difficult to separate in water. With the incorporation of modified materials, solid–liquid separation becomes easier, thereby enabling repeated recycling.

### 3.3 Adsorption studies

**Fig. 5:**
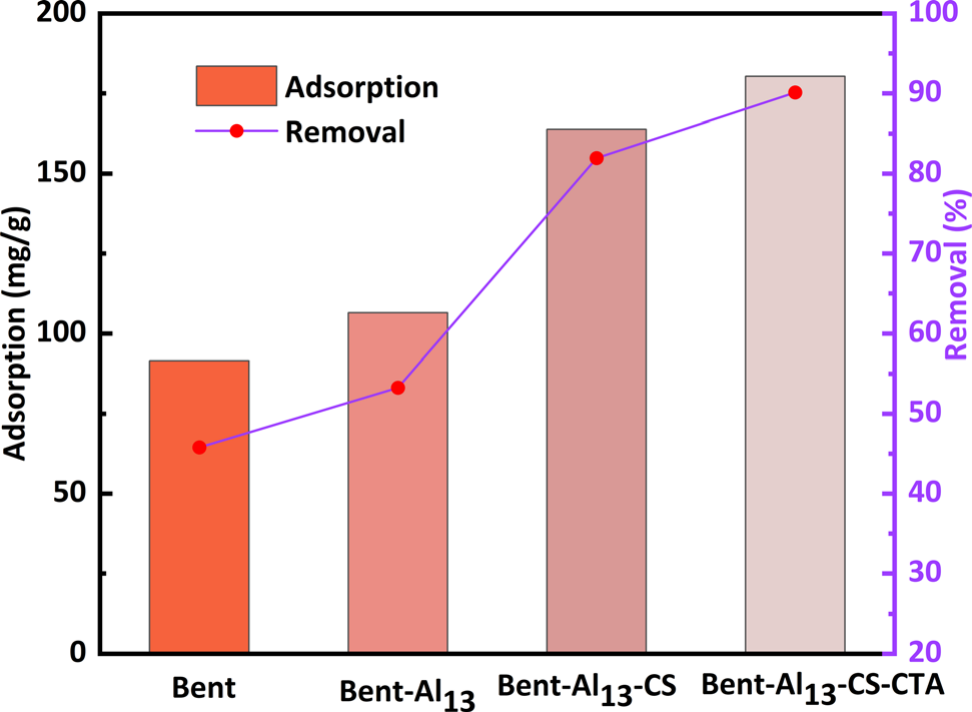
Comparison of the adsorption capacity and removal rate of bent, bent-Al_13_, bent-Al_13_-CS and bent-Al_13_-CS-CTA for CR (*C*_0_ = 400 mg L^−1^, dosage = 0.2 g L^−1^, *T* = 25 °C, *t* = 1 h, pH = 9).

Bent has an excellent adsorption capacity owing to its large inner surface area, which is a result of its structural properties. Even so, the removal of organic matter is relatively poor due to its hydrophilic nature, which repels organic matter. The removal rate of CR by bent is only 45.78%, as shown in Fig. 5. With the continuous addition of Al_13_, the layer spacing increased, which enhanced the removal effect, yet the hydrophilic properties were still maintained. After adding chitosan to bent-Al_13_, it becomes lipophilic and hydrophobic. The layer spacing is further expanded, and the dirt-holding and ion-exchange capacities are reinforced as CS molecular clusters replace interlayer cations. The material's surface adsorption capacity for organic contaminants was reinforced, and its removal rate reached 81.96% using the principle of similarity compatibility. The addition of citric acid led to an increase in the concentration of hydrogen ions on the surface of bent-Al_13_-CS. This increase provided additional adsorption sites for CR, resulting in an adsorption capacity of 180.32 mg g^−1^ and a removal rate of 90.16%.

**Fig. 6:**
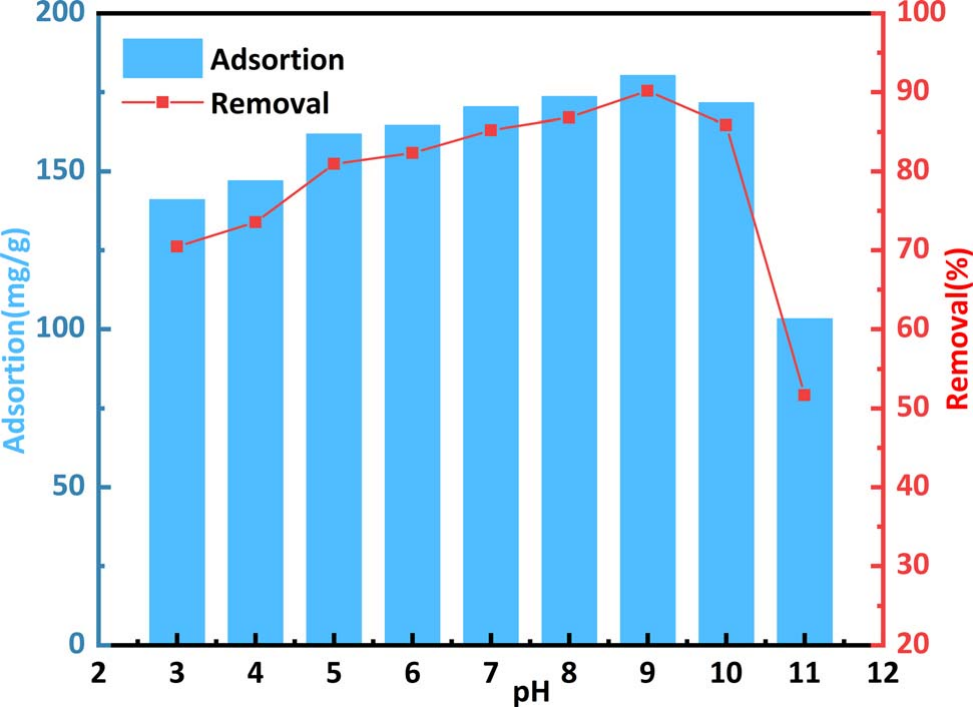
Effect of pH on the remove of CR by bent-Al_13_-CS-CTA (*C*_0_ = 400 mg L^−1^, dosage = 0.2 g L^−1^, *T* = 25 °C, *t* = 1 h).

The impact of pH (3–12) on the adsorption of a CR solution by bent-Al_13_-CS-CTA at 25 °C is depicted in Fig. 6. There are numerous active sites on the surface of the adsorbent, to which the dye molecules are attached through electrostatic force and chelation.^36^ Because of its influence on the ionization process of the dye molecules and the surface binding site of bent-Al_13_-CS-CTA, the pH of the solution was found to have a significant effect on dye uptake.

At lower pH levels, excessive H^+^ ions compete with each other and with H^+^ ions on the adsorbent for anionic dye molecules, thereby reducing their absorption. At higher pH values, anionic dyes and OH^−^ ions compete with each other for cationic adsorbents, resulting in a significant decrease in the removal rate.^37^ This phenomenon may also be attributed to OH^−^ ions destroying the structure of bent-Al_13_-CS-CTA. Within a pH range from 3 to 9, there is an improvement in removal rate from 70.54% to 90.16%. Considering the adsorption capacity and removal rate, it was found that the adsorption effect was better when the pH value of the CR solution was 9. The adsorption capacity was measured at 180.32 mg g^−1^, with a removal rate of 90.16%. It should be noted that throughout subsequent exploratory processes related to adsorption, solution pH remained constant.

**Fig. 7:**
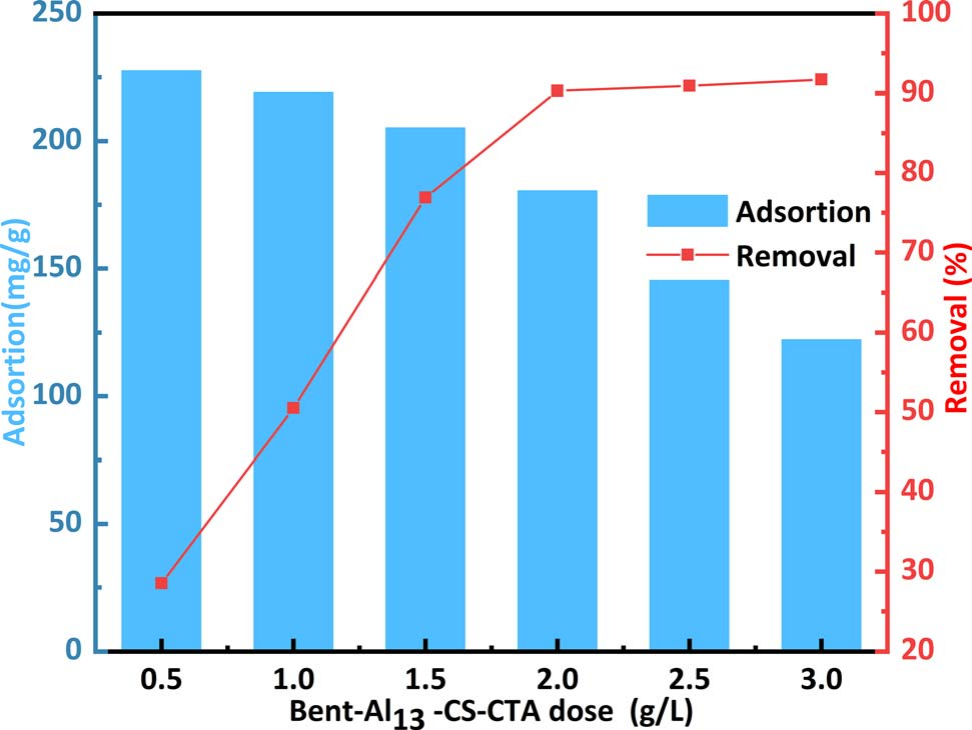
Effect of dosage on the remove of CR by bent-Al_13_-CS-CTA (*C*_0_ = 400 mg L^−1^, *T* = 25 °C, *t* = 1 h, pH = 9).

Fig. 7 shows how the dosage of the adsorbent affects the adsorption capacity and removal rate of CR varying dosages play a crucial role in maximizing cost efficiency and benefits within the dye adsorption system.^38^ When applying 0.5 g L^−1^ of the bent-Al_13_-CS-CTA, the CR adsorption capacity reached 227.6 mg g^−1^, resulting in a removal percentage of only 28.5%. The adsorption capacity declined as the dosage of bent-Al_13_-CS-CTA increased, yet the rate of dye removal grew over time. The usage of a 2 g L^−1^ dose of bent-Al_13_-CS-CTA led to the ultimate achievement of a maximum equilibrium of 90.3%. Because the dye concentration in solution was fixed, bent-Al_13_-CS-CTA at a dose of 2 g L^−1^ finally produced a maximum equilibrium of 90.3%. As a result, an overabundance of adsorbent causes competition amongst the adsorbents' active sites, which lowers the quantity of dyes absorbed per unit surface area. Consequently, 2 g L^−1^ of bent-Al_13_-CS-CTA was chosen as the dosage for CR elimination.

A larger adsorption capacity results from the adsorption sites being fully available for CR at a low dose of adsorbent. Though most of the low-energy adsorption sites were occupied first at high adsorbent dosage.^39^ As a result, there are fewer high-energy adsorption sites available, which lowers adsorption capacity.^40^ Additionally, there is a greater chance of collisions and agglomeration between solid particles with higher adsorbent doses. This ultimately results in a reduction of the overall surface area and the CR adsorption capacity of the bent-Al_13_-CS-CTA.

### 3.4 Adsorption isotherm

The adsorption isotherm reflects the relationship between the equilibrium concentration (*C*_e_) of the residual dye in the solution and the dye adsorption (*q*_e_) per unit weight of the adsorbent at different temperatures. Adsorption isotherms were investigated using the Langmuir and Freundlich models.^41^ Its linearized mathematical equation can be expressed as follows:3
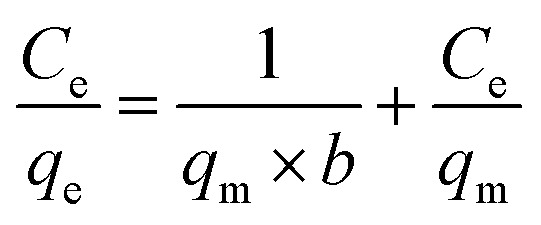
4
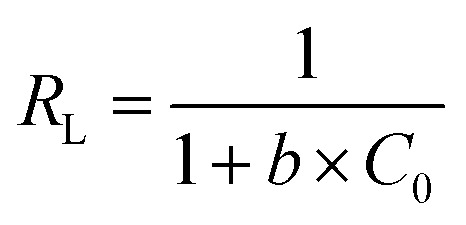
where *q*_m_ (mg g^−1^) is the theoretical estimation of maximum adsorption capacity and *b* (L mg^−1^) is the Langmuir constant. The linear plots of *C*_e_/*q*_e_ and *C*_e_ may be used to compute the intercept and slope, respectively, to find the Langmuir constant. *R*_L_ is a dimensionless constant referred to as the separation factor.

The Freundlich model for the adsorption isotherm is based on an empirical equation that assumes the surface of the adsorbent is heterogeneous and that the adsorption capacity depends on the concentration of the adsorbate. The mathematical equation for the linearized Freundlich model is represented as:5
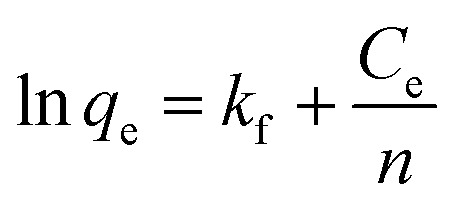
where exists a correlation between the adsorption intensity and capacity and the Freundlich constants, *k*_f_ and 1/*n*. They can be estimated by locating the intercept of the plot of ln *q*_e_ and ln *C*_e_.

**Fig. 8:**
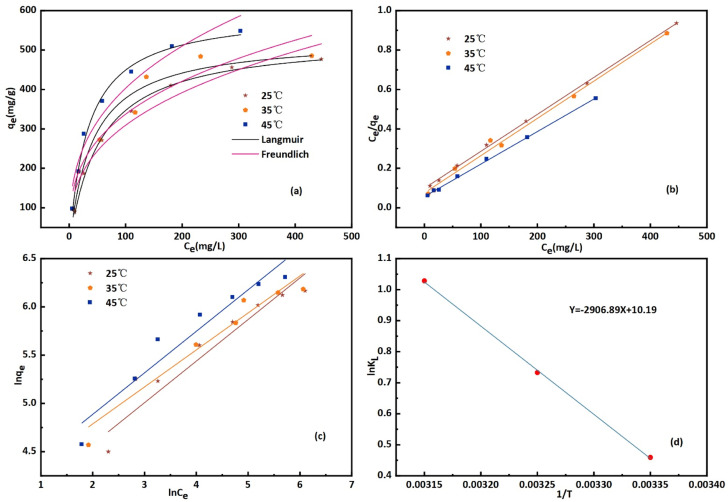
Plot of (a) adsorption isotherm, (b) Langmuir isotherms, (c) Freundlich isotherms and (d) thermodynamic parameters for the adsorption of CR onto bent-Al_13_-CS-CTA (*C*_0_ = 200, 400, 600, 800, 1000, 1200, 1400 ppm, dose = 0.2 g L; *T* = 25 °C, 35 °C, 45 °C; *t* = 1 h; pH = 9).

This phenomenon occurs due to the increase in diffusion rate of CR molecules at higher temperatures, while the viscosity of the solution decreases at higher temperature.^42^ For all molecules in both phases, mass transfer resistance is significantly influenced by the initial concentration. The CR molecule rapidly crosses the boundary layer through mass transfer and then diffuses into the internal voids of the bent-Al_13_-CS-CTA. At high temperatures, the CR molecule can gain additional energy to overcome resistance to transfer and form chemical bonds with the adsorption site of the bent-Al_13_-CS-CTA. The Langmuir and Freundlich isotherms at different temperatures (25 °C, 35 °C, 45 °C) are shown in Fig. 8(b) and (c), respectively.

Different Freundlich and Langmuir isotherm parameters were calculated from the intercept and slope of the linear equations and are summarized in Table 2. Freundlich and Langmuir were also fitted with nonlinear equations, as illustrated in Fig. 8(a). The Langmuir model outperformed the Freundlich model in both linear and nonlinear fitting, with an *R*^2^ value exceeding 0.99. The maximum adsorption capacity increased from 476.8 to 548.25 mg g^−1^ as the temperature increased from 25 °C to 45 °C. In addition, the values of the separation factor, *R*_L_, were between 0 and 1 at the three temperatures studied. This confirms that the adsorption of CR on bent-Al_13_-CS-CTA was favourable. In addition, the 1/*n* value at all three temperatures was less than 1.0, indicating that bent-Al_13_-CS-CTA was suitable for adsorbing CR over the entire range of study concentrations.^43^

**Table 2:** Langmuir and Freundlich isotherm constant at different temperatures

T (°C)	Langmuir isotherm constants					Freundlich isotherm constants			
	*b* (L mg^−1^)	*q*_m_ (mg g^−1^)	*R*_L_	Linear *R*^2^	Nonlinear *R*^2^	*K*_f_ (mg g^−1^) (L mg^−1^)	1/*n*	Linear *R*^2^	Nonlinear *R*^2^
25	0.0189	476.8	0.036–0.209	0.999	0.996	41.07	0.43	0.922	0.951
35	0.0251	485.31	0.023–0.166	0.993	0.949	55.76	0.38	0.926	0.924
45	0.0291	548.25	0.024–0.147	0.998	0.993	56.04	0.43	0.918	0.946

### 3.5 Thermodynamic parameters

Thermodynamic parameters were determined using the Van't Hoff equation:6
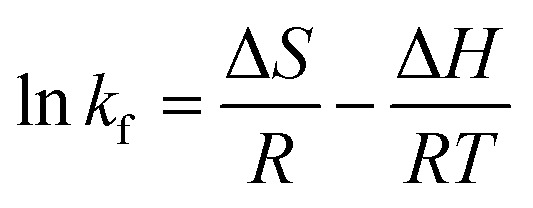

7 *G* = −*RT* ln *k*_f_

8
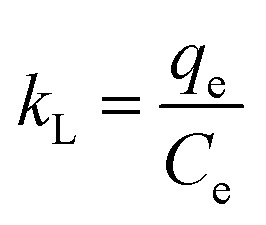
where *K*_L_ is the distribution coefficient, the changes in entropy, enthalpy, and free energy are represented by Δ*S*, Δ*H*, and Δ*G*, the adsorbent dose is given by *m* (g L^−1^), the equilibrium concentration is represented by *C*_e_ (mg L^−1^), the absolute temperature in *K* is given by *T*, and the gas constant is 8.314 J (K^−1^ mol^−1^) (Table 3).

**Table 3:** Thermodynamic parameters for the adsorption of CR onto bent-Al_13_-CS-CTA

Δ*G* (kJ mol^−1^)			Δ*H* (kJ mol^−1^)	Δ*S* (kJ (mol^−1^ K^−1^))
298.15 K	308.15 K	318.15 K	24.17	0.08
−1.14	−1.88	−2.72		

By calculating the slope and intercept of the linear plot of ln *K*_L_ and 1/*T* (Fig. 8(d)), one may determine the values of Δ*H* and Δ*S*. While the Δ*G* values exhibited negativity, positive values of Δ*H* and Δ*S* were observed at 24.17 kJ mol^−1^ and 0.08 kJ mol^−1^ K^−1^, respectively, as depicted in Table 4. The adsorption of CR solution indicated an endothermic process due to the positive value of Δ*H*, while the positive value of Δ*S* suggested an increase in disorder at the solid–liquid interface.^44^ The Δ*G* value was found to decrease from −1.14 kJ mol^−1^ at 25 °C to −2.72 kJ mol^−1^ at 45 °C. The fact that Δ*G* is negative suggests that CR can indeed adsorb spontaneously onto bent-Al_13_-CS-CTA. The Δ*G* value drops as temperature rises, suggesting that adsorption is more advantageous at higher temperatures.^45^

**Table 4:** Adsorption kinetic parameters

*C*_0_ (mg L^−1^)	400	600	800
Experimental value *q*_e_ (mg g^−1^)	187.76	272.6	345.4

Pseudo-first-order			
*k*_1_ (1 min^−1^)	0.05	0.033	0.031
Calculated value *q*_m_ (mg g^−1^)	75	109	198
Linear *R*^2^	0.881	0.828	0.855
Nonlinear *R*^2^	0.714	0.693	0.579

Pseudo-second-order			
*k*_2_ (g (mg^−1^ min^−1^))	0.00227	0.00087	0.00048
Calculated value *q*_m_ (mg g^−1^)	189.62	274.36	349.84
Linear *R*^2^	0.999	0.997	0.997
Nonlinear *R*^2^	0.926	0.897	0.839

Intra-particle diffusion			
*K*_i_ (mg (g^−1^ min^−0.5^))	4.169	7.833	12.41
*C* (mg g^−1^)	144	186	207
*R* ^2^	0.893	0.908	0.931

### 3.6 Adsorption kinetics

A crucial aspect of adsorption system design is the estimation of the velocity of the adsorption process, which is determined by the system's kinetics. The chemical and physical properties of the adsorbent determine the adsorption kinetics, which, in turn, affect the adsorption mechanism. Good correspondence between the kinetic and experimental data explains the kinetic mechanism. The adsorption constants can be calculated using the pseudo-second-order mechanism, pseudo-first-order mechanism, and the intra-particle diffusion model.^46^

The integrated form of the pseudo-first-order equation, pseudo-second-order, and intra-particle diffusion model^47^ is represented as:9
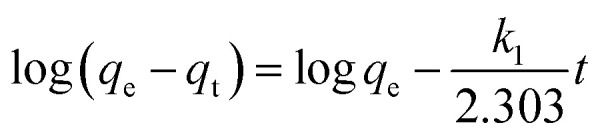
10
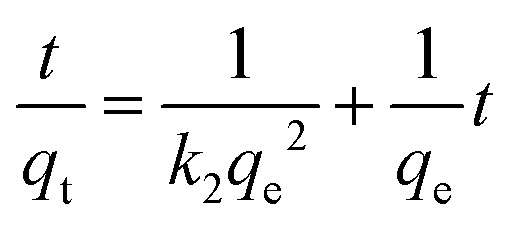

11 *q*_t_ = *k*_i_*t*^0.5^ + *C*

where *k*_1_ (1 min^−1^) is the pseudo-first-order rate constant, *q*_t_ (mg g^−1^) represents the dye uptake at time *t*, and *q*_e_ is the equilibrium adsorption capacity (mg g^−1^). *k*_2_ is the pseudo-second-order rate constant (g (mg^−1^ min^−1^)). *K*_i_ is the rate constant (mg (g^−1^ min^−0.5^)) and C (mg g^−1^) is the boundary layer thickness constant.

**Fig. 9:**
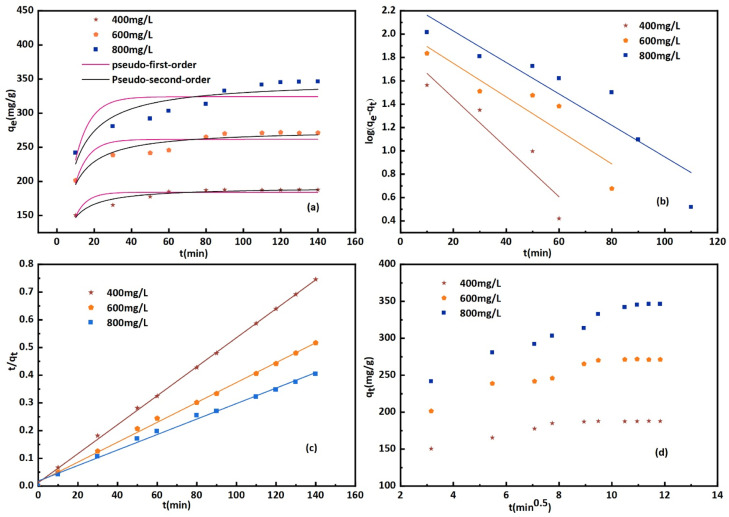
Plot of (a) kinetic data, (b) pseudo-first-order model, (c) pseudo-second-order model and (d) intra-particle diffusion model for the adsorption of CR onto bent-Al_13_-CS-CTA (*C*_0_ = 400, 600, 800 mg L; *t* = 10, 30, 50, 60, 80, 90, 110, 120, 130, 140 min; dose = 0.2 g L^−1^, *T* = 25 °C, pH = 9).

The impact of time and initial concentration on CR absorption is illustrated in Fig. 9(a). The adsorption kinetics were examined at dye concentrations of 400, 600, and 800 mg L^−1^. The experiment was conducted in a stirrer at 600 r min^−1^ and 25 °C. The increased surface area and quantity of micropores could explain the faster attainment of adsorption equilibrium.^48^ In the initial stage, there are numerous vacant adsorption sites on the surface of the modified bentonite, leading to a rapid adsorption rate and a quick increase in adsorption capacity. The low concentration of the CR solution reached adsorption equilibrium approximately one hour after the adsorption process began. Over time, the active site becomes gradually saturated with CR molecules, which causes a decrease in the adsorption rate and a delay in reaching equilibrium. Additionally, most of the active sites in bent-Al_13_-CS-CTA composites are located on the outer surface, making it easy for adsorbates to access them.^49^ Furthermore, the absorption of dye increased as the initial concentration increased. The adsorption capacity reached 345.4 mg g^−1^ when the adsorption equilibrium was achieved at a concentration of 800 mg L^−1^.

Pseudo-first-order and pseudo-second-order functional relationships are depicted in Fig. 9(b) and (c), respectively. Based on the intercept and slope of the fitting line, as well as the pseudo-first-order and pseudo-second-order nonlinear fitting curves shown in Fig. 9(a), the values of *k*_1_, *k*_2_, and *q*_e_ can be estimated. The results are presented in Table 4. The pseudo-second-order linear equation has an *R*^2^ value of 0.99, which is higher compared to the pseudo-first-order model.^50^ Although the *R*^2^ value for the nonlinear fit did not reach 0.99, it still outperformed the first-order nonlinear model. The pseudo-second-order model is more consistent with adsorption kinetics. Intra-particle diffusion model adsorbed the transportation of the adsorbed pollutants from the aqueous media to the fabricated adsorbent. Fig. 9(d) displays the *q*_t_ and *t*^0.5^ plots for three different dye concentrations. Intra-particle diffusion can be roughly divided into three stages, the first stage is instantaneous adsorption or external surface adsorption, the second stage is the gradual adsorption rate to the limit, and the third stage is the final equilibrium. The data shows a low correlation coefficient, and the line does not intersect the origin, suggesting that the primary rate-limiting factor of the dynamic process is not intra-particle diffusion.^51^ The mechanism of adsorption of CR by bent-Al_13_-CS-CTA was complex, and other factors such as boundary layer effects could also affect adsorption. The performance of the adsorption system was organized by a variety of operating organisms. The outcomes of the models verified that the pseudo-second-order kinetics best fit over the other kinetics adsorption models, Additionally, the experimental adsorptive values *q*_e_ correlated to the theoretically calculated value *q*_m_, indicating the validity of the results.

### 3.7 Desorption studies

Reusability and high adsorption capacity are essential. These properties are crucial for reducing the total cost of adsorbents. The desorption and regeneration processes of CR-adsorbed bent-Al_13_-CS-CTA were studied for five consecutive cycles. Mix the recovered sorbent with a 0.1 M HCl solution and shake for half an hour at room temperature. Rinse the recovered sorbent with water until the eluate is neutral. Since drying in the oven, the recovered adsorbent is reintroduced into service for the next adsorption cycle.

**Fig. 10:**
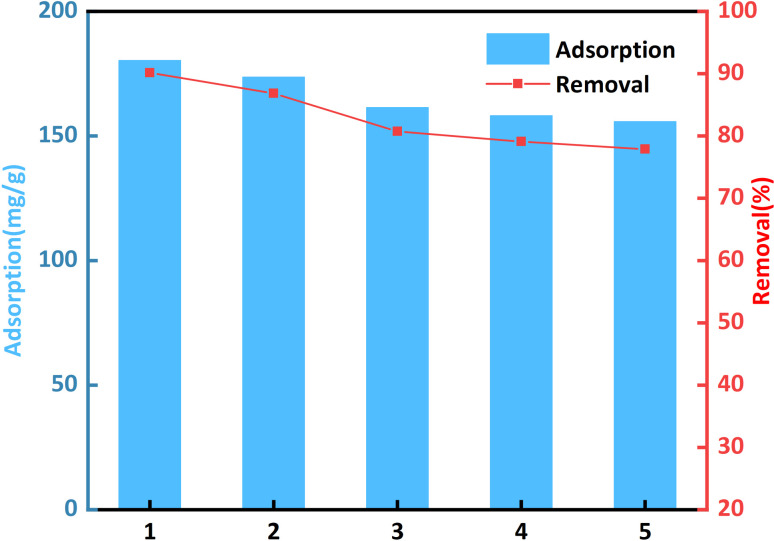
Effect of desorption cycles on CR removal (*C*_0_ = 400 mg L^−1^, dosage = 0.2 g L^−1^, *T* = 25 °C, *t* = 1 h, pH = 9).

Fig. 10 shows that after five cycles, the adsorption capacity of CR was 155.79 mg g^−1^ and the removal rate was 77.89%. This may be due to the predominance of the adsorbed CR dye molecule in pore size channels, a tiny amounts of chelation with citric acid, and hydrogen bonding. These factors result in a slight reduction in adsorption capacity and removal rate. Bent-Al_13_-CS-CTA, on the other hand, works well as an eco-friendly adsorbent for treating wastewater.

## 4. Conclusion

In this study, bent-Al_13_-CS-CTA, an exceptionally effective adsorbent for the removal of CR in aqueous solutions, was successfully synthesized. Due to its porous structure and large specific surface area, the resulting adsorbent offers numerous pathways for the rapid adsorption of CR dyes. The pH is 9, the amount of adsorbent is 0.2 g, the contact time is 1 hour, the initial concentration is 1400 mg L^−1^, and the maximum adsorption capacity can reach 476.8 mg g^−1^ at room temperature. Through fitting kinetic, thermodynamic linear, and nonlinear equations, it is finally established that the adsorption process conforms to the Langmuir isotherm model and the pseudo-second-order kinetic model. In addition, a recycling study was conducted, and after five repeated uses, the removal rate remained as high as 77.89%. It is very effective at removing CR and is environmentally friendly. Therefore, it may be suitable for the elimination of harmful substances and unwanted anionic dyes from industrial-scale treated wastewater.

## Author contributions

Methodology: Hanjie Chen and Shuyang Chen. Formal analysis, investigation: Mei Zhang. Data curation, visualization, and writing – original draft and validation: Hanjie Chen. Funding acquisition: Ying Fang. Writing – review & editing: Hanjie Chen and Ying Fang.

## Conflicts of interest

No conflict of interest exists in the submission of this manuscript, and the manuscript is approved by all authors for publication. I would like to declare on behalf of my co-authors that the work described was original research that has not been published previously, and is not under consideration for publication elsewhere, in whole or in part. All the authors listed have approved the manuscript that is enclosed.

## Supplementary Material
