# Correction: Study adsorbents based on bent-Al_13_-CS-CTA and its application to the removal of CR from wastewater

**DOI:** 10.1039/d5ra90066b

**Published:** 2025-05-30

**Authors:** Hanjie Chen, Mei Zhang, Shuyang Chen, Ying Fang

**Affiliations:** a Materials Science and Engineering Department, Nanjing Tech University Nanjing 210000 China powder8817@njtech.edu.cn

## Abstract

Correction for ‘Study adsorbents based on bent-Al_13_-CS-CTA and its application to the removal of CR from wastewater’ by Hanjie Chen *et al.*, *RSC Adv.*, 2024, **14**, 13817–13826, https://doi.org/10.1039/D4RA00197D.

The authors regret that the email address associated with affiliation *a* was shown incorrectly in the original article. The corrected email address is as shown here.

The Royal Society of Chemistry apologises for these errors and any consequent inconvenience to authors and readers.

